# Lipid deprivation amplifies type I IFN responses in monocytes through prenylation: insights from familial combined hypolipidemia type 2

**DOI:** 10.1186/s12967-025-07448-5

**Published:** 2025-11-25

**Authors:** Alessandra Pinzon Grimaldos, Ilenia Pacella, Tiziano Giacomelli, Simone Bini, Alessia Di Costanzo, Ilenia Minicocci, Laura D’Erasmo, Giuseppe Pietropaolo, Valerio Licursi, Marcello Arca, Silvia Piconese

**Affiliations:** 1 Department of Translational and Precision Medicine, Sapienza University of Rome, Rome, Italy; 2 IRCCS Neuromed, Pozzilli, Italy; 3 Institute of Molecular Biology and Pathology (IBPM), National Research Council (CNR) of Italy c/o Department of Biology and Biotechnology “C. Darwin”, Sapienza University of Rome, Rome, Italy; 4 Laboratory Affiliated to Istituto Pasteur Italia – Fondazione Cenci Bolognetti, Rome, Italy; 5 Present Address: Department of Surgery, Sapienza University of Rome, Rome, Italy; 6 Present Address: Neomatrix Biotec, Castelromano, Italy

**Keywords:** Immunometabolism, Cholesterol, Interferon signature, Isoprenoids

## Abstract

Familial Combined Hypolipidemia (FHBL2) is a genetic disorder caused by loss-of-function mutations in the Angiopoietin-like 3 (*ANGPTL3*) gene. FHBL2 subjects exhibit hypolipidemia and protection from atherosclerotic cardiovascular diseases. Here, we explored the hypothesis that immunometabolic events contribute to this atheroprotective phenotype. To this aim, circulating monocytes from FHBL2 subjects and controls were profiled through gene expression and phenotypic analysis in vivo and ex vivo. In parallel, immune responses were analyzed in monocytes that were lipid-deprived in vitro. In FHBL2 subjects, leukocytes exhibited lower content of intracellular lipids, together with a spontaneous type I IFN signature in vivo and higher sensitivity to IFN stimulation ex vivo. Lipid restriction in vitro was sufficient to recapitulate the higher IFN sensitivity in monocytes, while activating the mevalonate and isoprenoid synthetic pathway. These two events were linked, since a prenylation inhibitor reverted the high IFN response under lipid deprivation. Finally, we found that lipid restriction repressed, in a prenylation- and IFN-dependent fashion, the production of the inflammatory and proatherogenic cytokine IL-1β, and suppressed mitochondrial metabolism, which is a known trigger for inflammasome activation. In summary, we uncovered a novel immunometabolic mechanism linking lipid deprivation in monocytes, isoprenoid synthesis, enhanced IFN response, and IL-1β control. This circuit may provide an immunometabolic basis for the protection from atherosclerotic diseases in hypolipidemic subjects.

**Supplementary Information:**

The online version contains supplementary material available at 10.1186/s12967-025-07448-5.

## Graphical Abstract

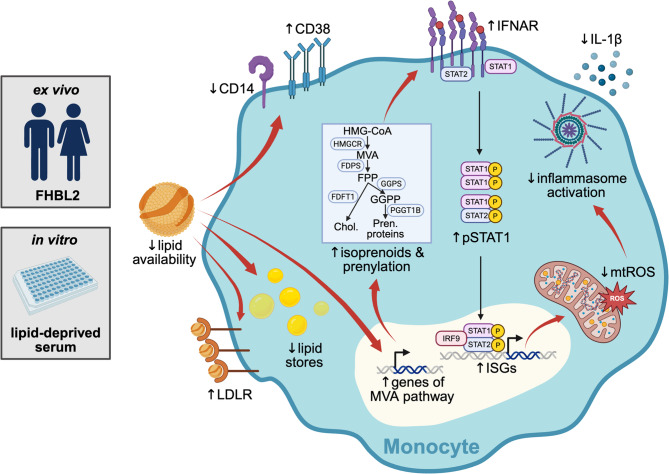

## Introduction

Innate immune responses converge into two major pathways: the induction of type I interferons (IFNs) and the activation of inflammasome.

Detection of foreign or self-derived nucleic acids by specific sensors activates the transcription factors IRF3 and IRF7, which drive the expression of IFNα/β. Once secreted, these cytokines bind to the IFNAR1 receptor on neighboring cells, triggering phosphorylation of STAT1 and STAT2 and the formation of the ISGF3 complex. ISGF3 promotes the transcription of numerous interferon-stimulated genes (ISGs) that establish an antiviral state [1]. IFNs are essential for controlling viral infections; however, they can also play pathogenic roles in autoimmune diseases characterized by an “interferon signature,” that is, constitutive ISG expression in blood and tissues [2].

Signals derived from cellular stress and damage, such as mitochondrial ROS release, activate the inflammasome—a cytosolic supramolecular complex that generates active caspase-1. Caspase-1, in turn, promotes the secretion of the mature form of the pro-inflammatory cytokine IL-1β. While inflammasome activation provides protection against microbial infections, it contributes to the pathogenesis of metabolic diseases and atherosclerosis [3].

An antagonistic relationship exists between the IFN and inflammasome pathways [4]. On one side, IL-1β limits IFN production through multiple mediators [5, 6]. On the other hand, IFN signaling suppresses inflammasome activation through several mechanisms, including induction of the anti-inflammatory cytokine IL-10 [7], inhibition of ROS generation [8], and the activity of the ISG cholesterol 25-hydroxylase *CH25H*, which reduces *Il1b* transcription [9].

Innate immune responses are tightly linked to cholesterol metabolism [10, 11]. Immune cells acquire cholesterol either through uptake of circulating lipoproteins via the LDL receptor (LDLR) or through de novo synthesis via the mevalonate pathway. This pathway also produces isoprenoids such as farnesyl diphosphate (FPP) and geranylgeranyl diphosphate (GGPP), which are required for the prenylation and activity of signaling proteins [12]. Excess cholesterol is stored in intracellular lipid droplets after esterification. Conversely, low cholesterol availability is sensed by sterol regulatory element-binding proteins (SREBPs), which promote both cholesterol uptake (via LDLR upregulation) and biosynthesis (through activation of the mevalonate pathway) [13].

The IFN and inflammasome pathways have opposing interactions with cholesterol metabolism. IFN signaling induces *CH25H* expression, represses SREBP activity, and reduces membrane cholesterol, thereby limiting viral entry. Conversely, genetic disruption of SREBP activity or cholesterol depletion in the endoplasmic reticulum (ER) membrane can spontaneously activate IFN responses [14]. In contrast, inflammasome activation is promoted by cholesterol overload: macrophages in the arterial wall internalize oxidized LDL, and cholesterol crystals trigger inflammasome activation, initiating atherogenesis [15].

Whether circulating lipoprotein levels influence intracellular cholesterol metabolism in immune cells—and thereby shape innate signaling—remains largely unexplored. Genetic dyslipidemias provide valuable models to investigate immunometabolism in vivo. In familial hypercholesterolemia (FH), circulating immune cells display increased IL-1 signaling and diminished IFN responses [16, 17]. Whether the opposite occurs in hypolipidemic conditions is unknown. Familial combined hypolipidemia type 2 (FHBL2, OMIM #605019) is caused by loss-of-function mutations in *ANGPTL3*, which increase lipolytic activity and markedly reduce plasma levels of all major lipoproteins [18]. Although extremely rare, FHBL2 confers striking protection against atherosclerotic cardiovascular disease [19], representing a unique human model to explore the immunometabolic consequences of lifelong severe hypolipidemia. ANGPTL3 has also emerged as a therapeutic target, with FDA- and EMA-approved antisense oligonucleotides and monoclonal antibodies, as well as additional agents in clinical development. Understanding how ANGPTL3 deficiency affects immune function is therefore of particular importance [20], both to clarify endogenous immunometabolic adaptations in FHBL2 and to anticipate potential immunological effects of pharmacological ANGPTL3 inhibition.

We recently reported an expansion of regulatory T cells in FHBL2, associated with a prenylation-dependent increase in immunoregulatory signaling in T cells, which may contribute to extrinsic suppression of inflammation in these individuals [21]. Here, we investigate the hypothesis that hypolipidemia in FHBL2 intrinsically rewires immunometabolic signaling within immune cells, promoting anti-inflammatory and anti-atherogenic pathways.

## Materials and methods

### Human subjects and samples

*ANGPTL3* homozygous (FHBL2) and heterozygous carriers, and age- and sex-matched controls, were recruited among the inhabitants of Campodimele town participating in a program of clinical characterization of *ANGPTL3* loss-of-function (LOF) mutation carriers [22]. The procedures of this program have been extensively described in previous reports [23–29] and were approved by the Institutional Review Board of Sapienza University of Rome under the approval code #4086.

The participants selected for the present investigation are a subpopulation of the larger cohort, having dietary intake, physical activity, smoking prevalence, and use of anti-inflammatory medications comparable to controls [29].

All subjects voluntarily participated in the study and written informed consent was obtained from all participants in accordance with the principles of the Helsinki Declaration.

Human peripheral blood was obtained from buffy coats of healthy donors (HDs) after written informed consent. Peripheral blood mononuclear cells (PBMCs) were isolated by density gradient centrifugation through Lympholyte (Cedarlane, Burlington, Canada). Cells were stored in freezing medium (DMSO 10% FBS) in liquid nitrogen until analysis.

### Monocyte isolation and culture

Monocytes were immunomagnetically isolated from buffy coats using CD14 Microbeads, human, according to manufacturer’s instruction (Miltenyi Biotec cat. 130-050-201). Purity was checked by flow cytometry and was higher than 85%.

Monocytes were cultured in 96-well Clear Round Bottom Ultra-Low Attachment Microplate (Corning cat. 7007) for 18 h at 37 °C in RPMI-1640 Dutch-modified medium containing 2 mM L-glutamine (Sigma-Aldrich), penicillin/streptomycin, nonessential amino acids, sodium pyruvate (EuroClone), and 50 µM 2-mercaptoethanol (Sigma-Aldrich). The medium was supplemented with one of the following sera: 10% Heat Inactivated serum from human male AB plasma (normal serum, NS) (SIGMA-Aldrich cat. H3667), 10% human lipoprotein deficient serum (LPDS) (SIGMA-Aldrich cat. LP4), or LPDS reconstituted with Low Density Lipoprotein from Human Plasma (LDL) (Invitrogen L3486) at 100 µg/mL. Both NS and LPDS contain a level of endotoxin < 3U/mL.

In some experiments, the following reagents were added: human IFN-α2b (Miltenyi Biotec cat. 130-093-875) 40.000 IU/mL, geranylgeranyl transferase inhibitor GGTi-2133 (Sigma-Aldrich cat. G5294) 30µM, or Anifrolumab (anti-IFNAR1) 2.5  µg/mL. After culture, monocytes were transferred in 96-well Clear Round Bottom tissue culture-treated plate (Corning cat. 3799) for subsequent analyses.

### Flow cytometry

To analyze intracellular lipid content, cells were first incubated for 20 min at 37 °C with Fixable Viability Dye eFluor780 (Thermo Fisher Scientific) plus Bodipy (BODIPY 505/515, 4,4-Difluoro-1,3,5,7-Tetramethyl-4-Bora-3a,4a- Diaza-s-Indacene; Thermo Fisher Scientific cat. D3921), a lipophilic fluorophore specific for neutral lipid stores such as intracellular lipid droplets. Then, cells were surface stained for 20 min at 4 °C with combinations of the following Abs: CD14 BV421 (BD cat. 563743), IFNAR1 PE (Invitrogen cat. MA5-23630), LDLR BV605 (BD cat. 745174), CD38 BV605 (BD cat. 740401), HLA-DR PE-CF594 (BD cat. 562304) and CD19/CD56/CD8/CD4 as exclusion markers. CD38 was chosen to identify monocytes ex vivo together with CD14, based on previous literature [30, 31].

For mitochondrial profiling, after viability dye and surface staining, cells were labelled with MitoTracker Deep Red (Thermo Fisher Scientific cat. M22426) 50 nM for 30 min at 37 °C, with TMRM (Thermo Fisher Scientific cat. M20036) 10 nM for 30 min at 37 °C, or with MitoSOX Mitochondrial Superoxide (Thermo Fisher Scientific cat. M36007) 10 µM for 20 min at 37 °C. The TMRM dye was used by applying a non-quenching mode, whereby depolarized cells display decreased fluorescence as evidenced by the minimal signal obtained with the depolarizing agent CCCP [32].

Intracellular staining was performed for 30 min at room temperature after fixation and permeabilization with the FOXP3/Transcription Factor Staining Buffer Set according to the manufacturer’s instructions (Thermo Fisher Scientific) using ISG15 PE (R&D Systems cat. IC8044P). In some experiments, cells were stimulated 18 h with human IFN-α2b (Miltenyi Biotec 130-093-875) 40.000 U/mL before staining.

For the analysis of STAT1 phosphorylation, cells were stimulated with IFN-α2b 40.000 U/ml (Miltenyi Biotec) for 15 min at 37 °C, then fixed and permeabilized using Cytofix Fixation Buffer (BD Biosciences, pre-warmed to 37 °C) and Perm Buffer III (BD Biosciences, pre-cooled to -20 °C), according to the instructions of the manufacturer. Finally, intracellular staining with Stat1 (pY701) Alexa Fluor 488 (BD cat. 612596) was performed for 30 min at room temperature in PBS 0.5% BSA.

To analyze cytokine production, cells were stimulated with lipopolysaccharide (LPS) 100 ng/mL and Protein Transport Inhibitor Cocktail (Thermo Fisher Scientific) for 4 h at 37 °C. After viability dye and surface staining, cells were fixed and permeabilized with BD Cytofix/Cytoperm Fixation/Permeabilization Solution Kit (cat. 554714) for 20 min at + 4 °C. Then, cells were incubated with combinations of the following Abs: anti-IL-1β Alexa Fluor 647 (BioLegend cat. 508207), IL-6 PE (BD cat.554545), TNF FITC (Miltenyi Biotec. Cat. 130-091-650).

Samples were acquired on the BD LSRFortessa cell analyzer (BD Biosciences) and analyzed with FlowJo software, version 10.0.8r1 (BD Biosciences). Samples with at least 100 events in the monocyte gate were included in the final analysis.

### RNA extraction, RNAseq and and real time PCR

For RNAseq, RNA was extracted using the RNeasy Plus Mini Kit Qiagen (Cat. 74134) from at least 4 × 10^6 PBMCs. RNA yield was determined using the Nanodrop 2000/2000c Spectrometer (Thermofisher). RNA-Seq libraries for mRNA sequencing were prepared with Truseq Stranded mRNA Prep Kit. All extracted RNA samples were quality-controlled for integrity with a 2100 Bioanalyzer system (Agilent Technologies) and sequenced on a NovaSeq 6000 System (Illumina). Raw data were processed for both format conversion and de-multiplexing by Bcl2Fastq v.2.20 of the Illumina pipeline. Read quality was evaluated using FastQC v.0.11.8 (Babraham Institute) tool. Then adapter sequences were removed with trimgalore v.0.6.6 from FASTQ sequences using the auto detection mode. Reads were mapped to the mouse Ensembl GRCh38 transcriptome index using salmon aligner (v.1.5.0) [33] with a decoy-aware transcriptome index with k-mers of length 31 and normalizing for local GC content. GENCODE Gene Set version 38 was used for gene annotation. Gene-level normalization and differential gene expression analysis were performed with Bioconductor [34] packages tximport v.1.19 and DESeq2 v.1.28 [35] using the R environment v.4.0. To check for outliers, a regularized log transformation was applied to the count data, and a principal component analysis was generated based on genes showing the highest variance across all samples. The Wald test was used for significance testing, and the resulting FDR P values were adjusted for multiple comparisons using the Benjamini and Hochberg method [36]. Genes were considered differentially expressed genes (DEGs) only at FDR < 0.05. Gene set enrichment analysis was performed with Bioconductor R package clusterProfiler v.4.2 [37] with annotation of Gene Ontology Database [38]. For the quantification of immune cell populations signatures, the ImSig [39] approach was employed as it offers a set of immune gene signatures derived from tissue transcriptomics data using a network-based deconvolution approach.

For quantitative real-time RT-PCR, RNA was extracted from cultured monocytes using RNeasy Plus Micro Kit Qiagen (Cat. 74034) and cDNA was obtained with Superscript VILO cDNA Synthesis Kit (Thermo Fisher Scientific). RT-PCR was performed to analyze the expression of selected genes using the following Taqman assays: *HMGCR* Hs00168352_m1, *FDFT1* Hs00926054_m1, *PGGT1B* Hs00270701_m1, *GGPS1* Hs01546492_g1, *FDPS* Hs01578769_g1, *MX1* Hs00895608_m1, *IFIT1* Hs03027069_s1, *ISG15* Hs01921425_s1. RT-PCR was performed in duplicates using the TaqMan Fast Advanced Master Mix (Thermo Fisher Scientific cat. 4444557). Sample values were normalized to the Ct value for *HPRT1* Hs02800695_m1 gene using the formula 2^−ΔCt.

### Statistics

The analysis was performed using Prism 10.1.1 (GraphPad). Data are presented as means ± SD. Two tailed Mann-Whitney test or 2way ANOVA with Šídák’s multiple comparisons test were applied in the analysis of not matched samples. Wilcoxon’s test, 1way ANOVA with Dunnett’s multiple comparison test or 2way ANOVA with Tukey’s multiple comparisons test were used to analyze matched samples. In some experiments, Student’s t test, 2-tailed and unpaired, was used. When parametric tests were applied, normality was verified with Kolmogorov-Smirnov and/or Shapiro-Wilk test. Spearman’s analysis was used to assess correlations. P values less than 0.05 were considered statistically significant.

## Results

### Lipid deprivation in monocytes promotes IFN response in vivo in FHBL2 and in vitro

**Fig. 1:**
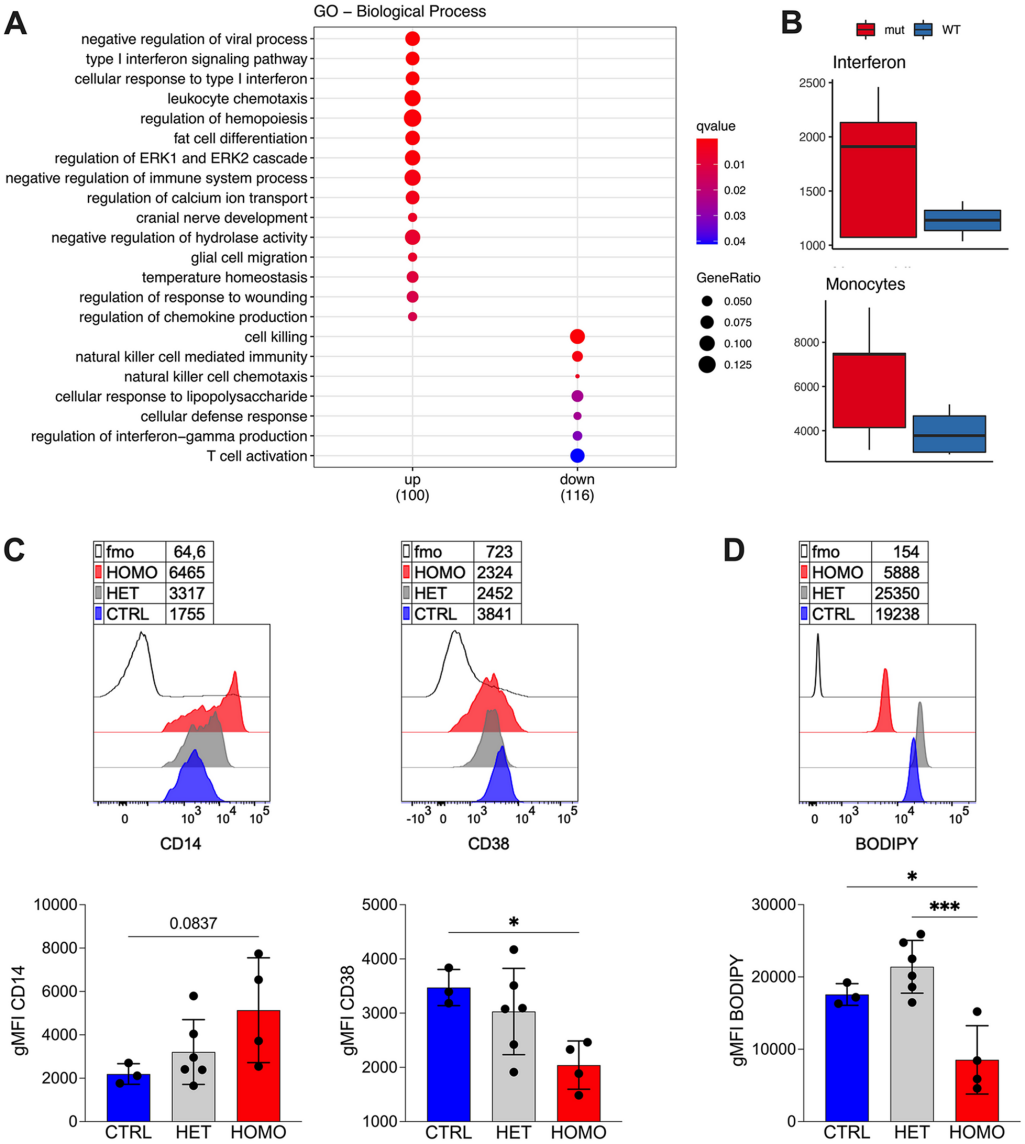
Interferon and monocyte signature upregulation in FHBL2 subjects. **A-B** PBMCs from FHBL2 homozygous subjects (*n* = 5) and age- and sex-matched non mutated controls (*n* = 4) were analyzed by bulk RNAseq analysis. (**A**) GO pathway analysis of the DEGs. (**B**) Enrichment of the “Interferon” and the “Monocytes” signatures of immune cell types and responses. **C-D** PBMCs of homozygous (HOMO, *n* = 4) and heterozygous (HET, *n* = 6) FHBL2 subjects and age- and sex-matched healthy controls (CTRL, *n* = 3) were analyzed by flow cytometry. Representative histogram overlays and cumulative analysis of surface CD38 and CD14 expression (**C**) and Bodipy content (**D**) are shown, as the geometric mean fluorescence intensity (gMFI), in gated CD14 + CD38 + monocytes. fmo, fluorescence-minus-one control. Only samples with > 100 events in the monocyte gate were included. Each dot is from a single subject. Bars represent means and SD. * *P* < 0.05, ***P < 0.001 by 1way ANOVA with Dunnett’s multiple comparisons test

To investigate the impact of hypolipidemia on circulating immune cells, we compared the gene expression profile of peripheral blood mononuclear cells (PBMCs) from FHBL2 homozygous individuals (*n* = 5) and non-mutated controls (*n* = 4). Consistent with previous data [29], FHBL2 individuals showed a marked reduction in circulating cholesterol, lipoproteins, and triglycerides (Table 1). Bulk RNA-seq analysis identified 261 differentially expressed genes (fold change >1.5, FDR < 0.10). Type I interferon (IFN) signaling pathways were significantly upregulated in FHBL2 samples (q < 0.01), whereas NK cell–related signatures were reduced (Fig. 1A, Suppl. Figure 1A–B). Despite some interindividual variability (Suppl. Figure 1C), genes annotated within the type I IFN signaling pathway (GO:0060337) were consistently upregulated in the FHBL2 cohort. Among immune cell signatures, NK-related genes were reduced (Suppl. Figure 1D), while both monocyte and interferon signatures were elevated in FHBL2-derived cells (Fig. 1B).

**Table 1:** Features of subjects enrolled for gene expression analysis

ID	Genotype	Sex	Age (years)	TC (mg/dl)	HDL (mg/dl)	LDL (mg/dl)	TG (mg/dl)
445	FHBL2 Homozygous	M	63	83	38	39	28
372	FHBL2 Homozygous	M	67	80	29	46	23
141	FHBL2 Homozygous	M	73	77	30	21	28
463	FHBL2 Homozygous	F	59	98	23	64	54
499	FHBL2 Homozygous	F	89	93	20	62,2	54
142	Control	F	62	218	77	129	62
398	Control	F	65	279	69	178	158
81	Control	M	71	155	64	74	86
570	Control	M	91	120	22	70,6	137

TC, total cholesterol; TG, triglycerides

To validate this observation, we next assessed monocyte frequency, phenotype, lipid content, and IFN responsiveness in a second cohort including FHBL2 homozygous and heterozygous subjects, as well as controls. Circulating monocytes were identified as shown in Suppl. Figure 2A. In this cohort, the lipid profile of homozygous subjects differed significantly from that of heterozygous individuals and controls (Table 2). Only a non-significant trend toward increased monocyte frequency was observed in homozygous FHBL2 subjects compared to the other groups (Suppl. Figure 2B), and monocyte frequency did not correlate with total cholesterol (TC), low-density lipoprotein (LDL), high-density lipoprotein (HDL), or triglyceride (TG) levels (Suppl. Figure 2C).

**Table 2:** Features of subjects enrolled for phenotypic analyses

ID	Genotype	Sex	Age (years)	TC (mg/dl)	HDL (mg/dl)	LDL (mg/dl)	TG (mg/dl)
445	FHBL2 Homozygous	M	63	83	38	39	28
372	FHBL2 Homozygous	M	67	80	29	46	23
141	FHBL2 Homozygous	M	73	77	30	21	28
1001	FHBL2 Homozygous	F	46	na	35,7	na	na
139	FHBL2 Heterozygous	F	59	190	63	109,4	88
148	FHBL2 Heterozygous	F	51	182	35	124,6	112
175	FHBL2 Heterozygous	M	46	181	47	116,8	86
399	FHBL2 Heterozygous	F	56	209	49	152,2	39
489	FHBL2 Heterozygous	M	60	202	79	115,2	39
514	FHBL2 Heterozygous	F	71	195	75	106,2	69
204	Control	F	70	265	54	195	95
526	Control	F	75	140	36	97,6	32
483	Control	F	76	182	59	96	136
46	Control	M	73	200	40	138,2	109
81	Control	M	71	155	64	74	86
914	Control	F	80	108	34	59,4	73

na, not available; TC, total cholesterol; TG, triglycerides

We then examined monocyte phenotype based on CD14 and CD38 expression, quantified as geometric mean fluorescence intensity (gMFI). CD14 expression was increased, while CD38 expression was reduced, in monocytes from FHBL2 homozygous subjects compared with controls, with heterozygous individuals displaying an intermediate phenotype (Fig. 1C). Intracellular lipid droplets, quantified by Bodipy staining, were significantly reduced in homozygous subjects compared with heterozygous individuals and controls (Fig. 1D). CD38 expression and intracellular lipid content showed a significant positive correlation with plasma levels of TC, LDL, HDL, and TG, whereas CD14 expression did not correlate with plasma lipid concentrations (Suppl. Figure 3). These findings suggest that CD38 expression and intracellular lipid load are directly influenced by the availability of circulating lipids. Supporting a potential immunometabolic link between monocyte phenotype and atheroprotection, CD14 expression tended to correlate inversely, and CD38 expression positively, with systolic blood pressure (Suppl. Figure 4).

**Fig. 2:**
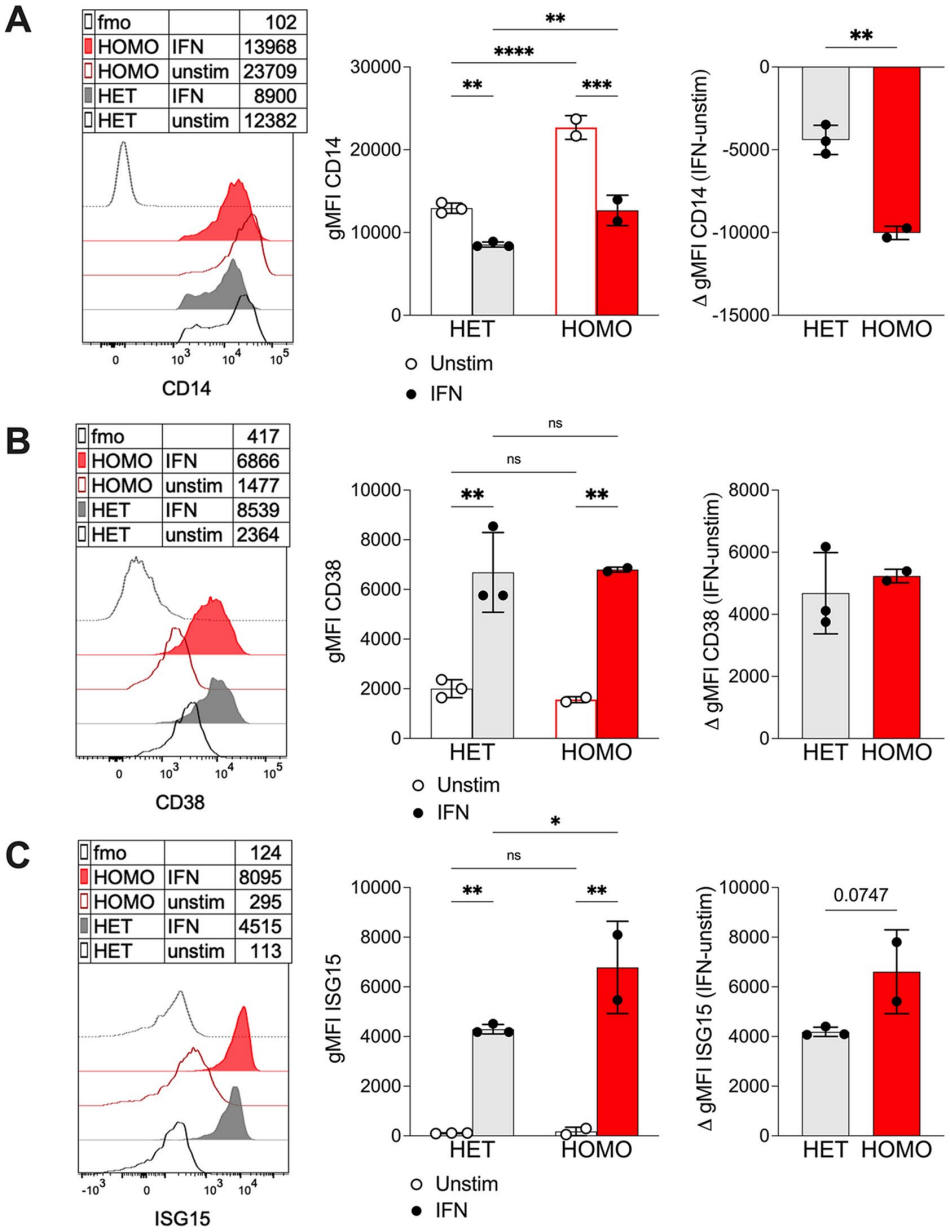
FHBL2 monocytes display a higher responsiveness to type I IFN. PBMCs of homozygous (HOMO, *n* = 2) or heterozygous (HET, *n* = 3) FHBL2 subjects were cultured 18 h in presence or not of type I IFN (40.000 IU/mL). The data show histogram overlays and cumulative analysis of the absolute gMFI, as well as Δ gMFI (calculated as the difference in the gMFI of IFN-stimulated and unstimulated cells), for the expression of CD14 (**A**), CD38 (**B**), and ISG15 (**C**). Only samples with > 100 events in the monocyte gate were included. Each dot is from a single subject. Bars represent means and SD. *P < 0.05, **P < 0.01, ***P < 0.001, ****P < 0.0001 by RM 2way ANOVA with Šídák’s multiple comparisons test. **P < 0.01 by unpaired T test, for the Δ gMFI analysis

To assess monocyte responsiveness to type I IFN, PBMCs from FHBL2 subjects were cultured for 18 h in the presence of recombinant IFN-α. Due to substantial IFN-induced cytotoxicity, this analysis was feasible only in samples from FHBL2 homozygous (*n* = 2) and heterozygous (*n* = 3) individuals in which more than 100 monocyte events remained after culture. IFN exposure reduced CD14 expression and increased CD38 expression in monocytes from both genotypes; however, the reduction in CD14 was significantly greater in homozygous individuals, indicating heightened IFN sensitivity (Fig. 2A–B). Direct measurement of IFN responsiveness revealed strong induction of the IFN-stimulated protein ISG15 in monocytes from both homozygous and heterozygous individuals, with significantly higher expression in homozygous subjects (Fig. 2C).

Taken together, these results indicate that FHBL2 is associated with a spontaneous type I IFN signature and that monocytes from FHBL2 individuals display reduced intracellular lipid content and enhanced IFN sensitivity ex vivo.

**Fig. 3:**
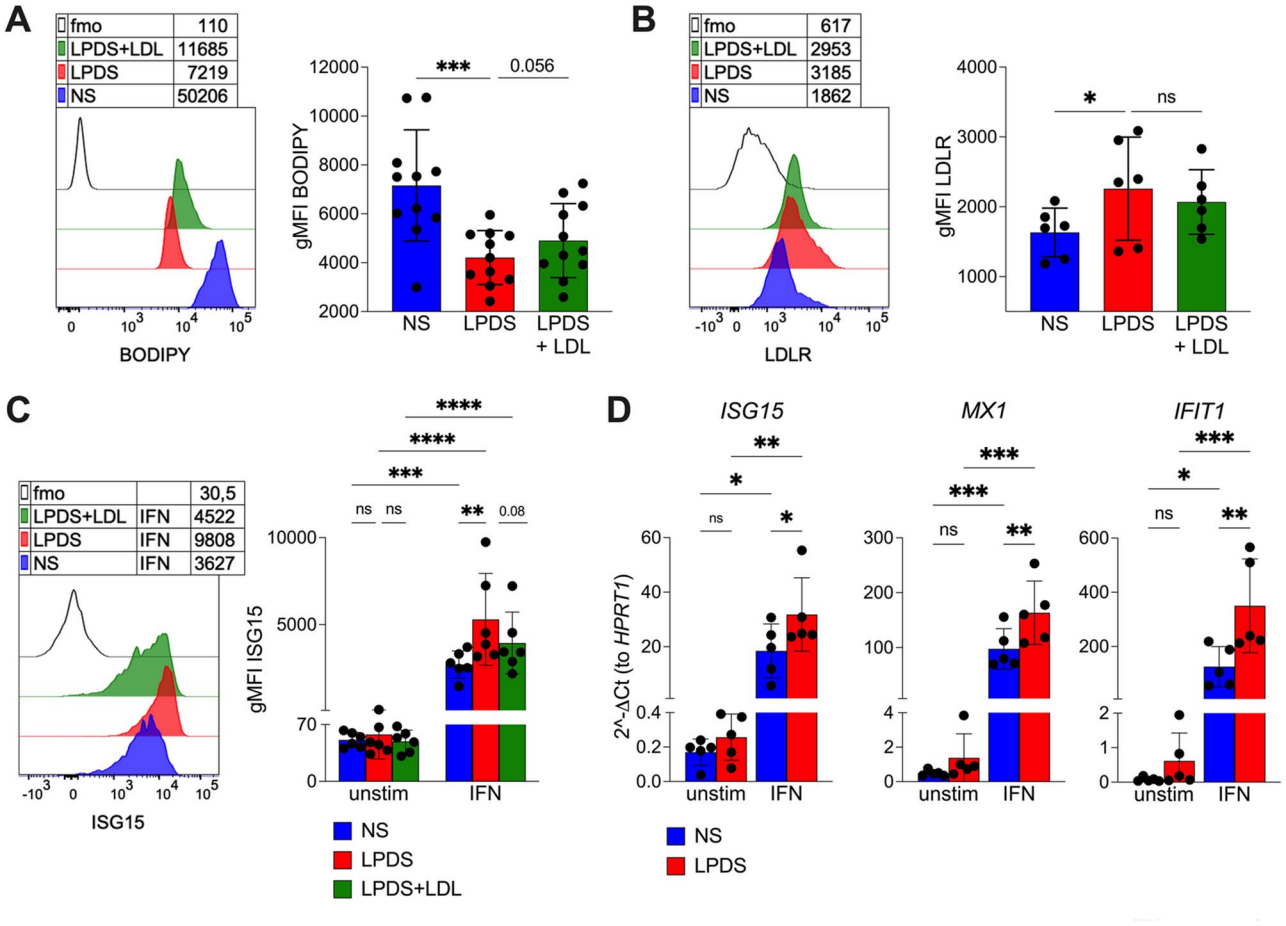
Monocytes deprived of lipids in vitro are more responsive to type I IFN. **A-B** Monocytes were enriched from PBMCs of healthy controls, and cultured 18 h in the presence of NS, LPDS, or LPDS + LDL. Then, cells were analyzed by flow cytometry. (**A**) Histogram and cumulative analysis of Bodipy labeling. Data are from 11 HD, pooled from 3 independent experiments. (**B**) Histogram and cumulative analysis of LDLR expression. Data are from 6 HD, from 2 independent experiments. Each dot is from a single HD. Bars represent means and SD. * *P* < 0.05, ***P < 0.001 by 1way ANOVA with Dunnett’s multiple comparisons test. **C** For ISG15 induction, recombinant IFN (40.000 IU/mL) was added in culture. Histogram and cumulative analysis of the ISG15 expression in absence (unstim) or presence of IFN. Data are from 6 HD, pooled from 2 independent experiments. **P < 0.01, ***P < 0.001, ****P < 0.0001, by 2way ANOVA with Tukey’s multiple comparisons test. **D** *ISG15*, *MX1*, *IFIT1* gene expression was analyzed by real-time RT-PCR in monocytes cultured in NS or LPDS, with or without IFN. Data are from 5 donors. Bars represent means and SD. *P < 0.05, **P < 0.01, ***P < 0.001, by 2way ANOVA

To determine whether lipid deprivation alone is sufficient to reproduce these features, we established a reductionist in vitro system in which monocytes isolated from healthy donors were cultured with normal serum (NS) or lipoprotein-depleted serum (LPDS), with or without LDL reconstitution. Lipid-deprived monocytes exhibited a significantly reduced intracellular lipid content by Bodipy staining, which was partially restored by LDL supplementation (Fig. 3A). Lipid deprivation also resulted in upregulation of surface LDLR expression (Fig. 3B). No spontaneous ISG15 expression was detected in lipid-deprived monocytes; however, these cells displayed heightened sensitivity to IFN-α stimulation, as shown by increased ISG15 induction, which was partially reversed by LDL repletion (Fig. 3C). Enhanced IFN responsiveness under lipid deprivation was further confirmed by increased expression of *ISG15*, *MX1*, and *IFIT1* mRNAs upon IFN stimulation (Fig. 3D).

### Mevalonate pathway is activated in monocytes under lipid restriction and promotes prenylation-dependent IFNAR1-STAT1 signaling

Given that the results described above indicated that lipid restriction enhances monocyte sensitivity to type I IFN, we next sought to identify the mechanistic link between these two phenomena. We previously demonstrated that lipid starvation induces activation of the mevalonate pathway and isoprenoid synthesis in human CD4⁺ T cells in vitro, thereby supporting cytokine signaling in a prenylation-dependent manner [21]. We therefore hypothesized that a similar mechanism underlies the enhanced IFN response observed in lipid-restricted monocytes.

**Fig. 4:**
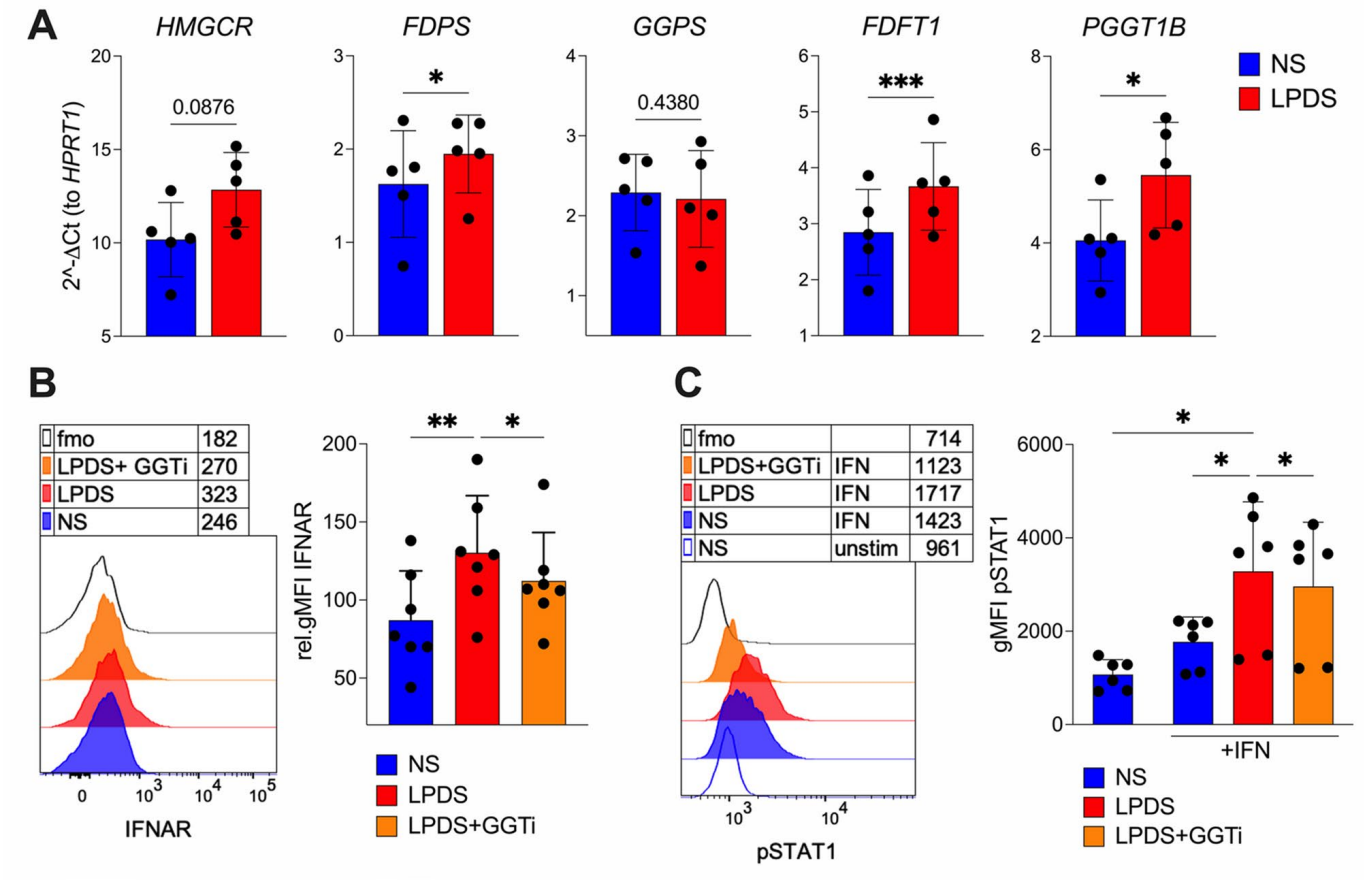
Lipid-deprived monocytes activate the mevalonate pathway and increase the early responsiveness to type I IFN in a prenylation-dependent manner. **A** Monocytes from healthy controls were cultured 18 hrs with NS or LPDS. The expression of *HMGCR*, *FDPS*, *GGPS*, *FDFT1* and *PGGT1b* genes was analyzed by real-time RT-PCR. Data are from 5 donors. Bars represent means and SD. *P < 0.05, ***P < 0.001, by paired T test. **B-C** Monocytes were cultured 18 hrs with NS, LPDS, or LPDS supplemented with geranyl-geranyl transferase inhibitor (GGTi 30 μM), then IFNAR expression was analyzed by flow cytometry. (**B**) Histogram overlay of IFNAR gMFI, and cumulative analysis of the relative gMFI (calculated by subtracting the respective fmo control for each sample), are shown. Data are from 7 donors from 2 independent experiments. (**C**) IFN (40.000 IU/mL) was added in the last 15 minutes of culture, then STAT1 phosphorylation was analyzed by flow cytometry. Histograms and cumulative analysis of gMFI of pSTAT1 are shown. Data are from 6 donors from 2 independent experiments. Bars represent means and SD. *P < 0.05, by 1way ANOVA with Dunnett’s multiple comparisons test

To test this, we measured the expression of genes encoding key enzymes in the mevalonate pathway, including 3-hydroxy-3-methylglutaryl-CoA reductase (*HMGCR*), farnesyl diphosphate synthase (*FDPS*), geranylgeranyl diphosphate synthase 1 (*GGPS1*), farnesyl-diphosphate farnesyltransferase 1 (*FDFT1*), and geranylgeranyltransferase type I subunit beta (*PGGT1B*). All genes except *GGPS1* were upregulated in monocytes cultured in LPDS (Fig. 4A). Notably, *HMGCR*, *FDFT1*, and *PGGT1B* also tended to be expressed at higher levels in PBMCs from FHBL2 subjects compared with controls (Suppl. Figure 5). These findings indicate that cholesterol depletion activates the mevalonate pathway, including the isoprenoid biosynthetic branch that supports protein prenylation.

To functionally assess the role of prenylation in regulating IFN sensitivity, we examined IFNAR expression and STAT1 phosphorylation in response to IFN stimulation in the presence or absence of a geranylgeranyl transferase inhibitor (GGTi). LPDS-treated monocytes showed increased surface expression of IFNAR, an effect that was significantly reduced by GGTi treatment (Fig. 4B). Consistent with this, lipid-deprived monocytes exhibited enhanced STAT1 phosphorylation following IFN stimulation, which was also reversed by prenylation inhibition (Fig. 4C). Together, these findings support a model in which reduced availability of exogenous lipids promotes mevalonate pathway activation and isoprenoid synthesis, thereby enhancing proximal IFN signaling in monocytes in a prenylation-dependent manner.

### Lipid deprivation suppresses IL-1β production and mitochondrial activity in vitro

Type I IFNs negatively regulate IL-1β production in human monocytes in vitro [40], and monocytes from IFN-treated patients produce less IL-1β ex vivo [7]. We therefore tested the hypothesis that the enhanced IFN response induced by lipid restriction suppresses IL-1β production. To this end, monocytes were cultured under conditions of lipid restriction or lipid replenishment, and the intracellular levels of inflammatory cytokines directly (IL-1β) or indirectly (TNF, IL-6) linked to inflammasome activation were measured.

**Fig. 5:**
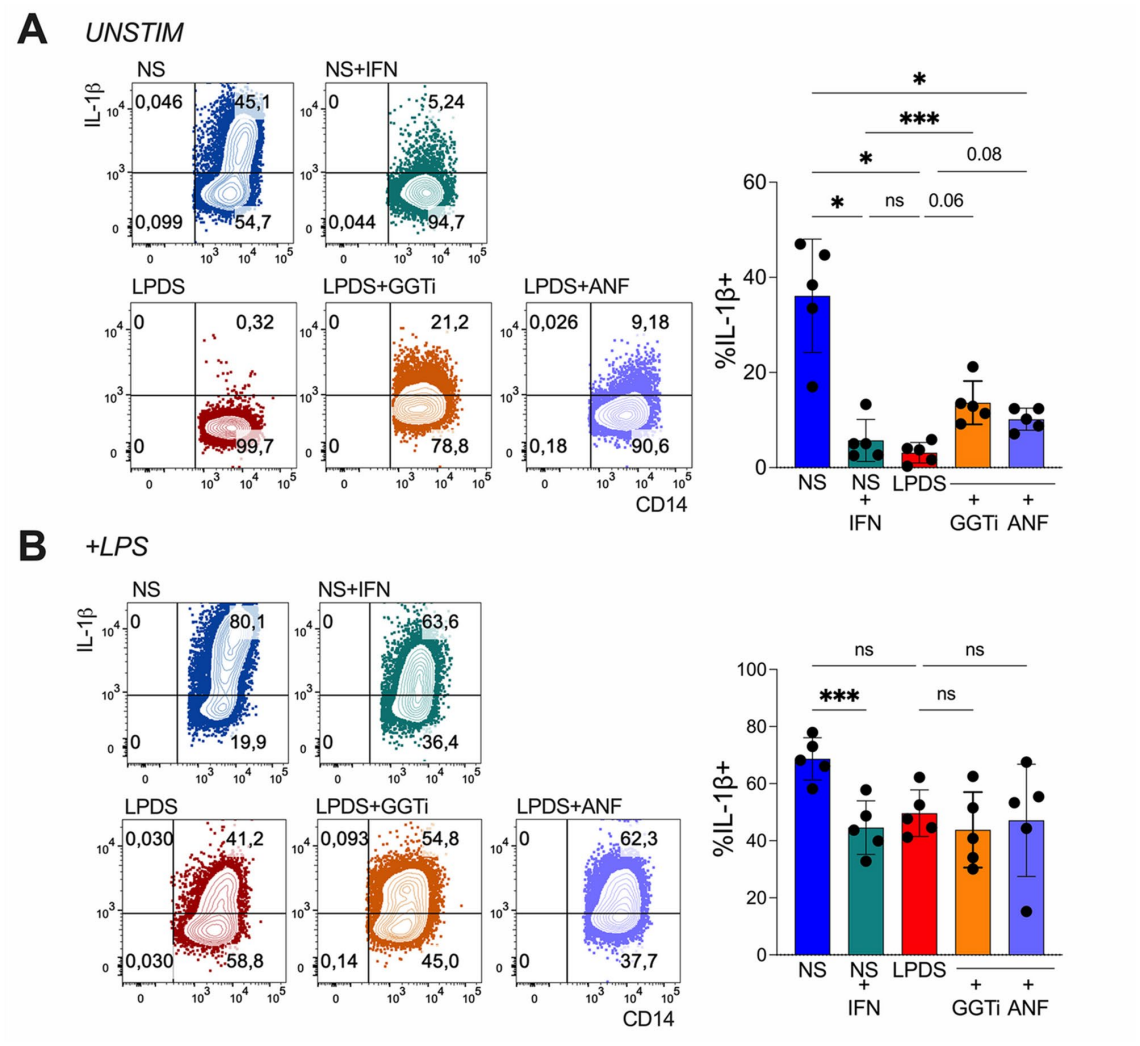
Lipid-deprived monocytes produce less IL-1β in a GGTi- and IFN-dependent fashion. Monocytes, cultured 18 h with NS or LPDS, were left unstimulated or stimulated with LPS (100 ng/mL), IFN (40.000 IU/mL), GGTi (30 µM) or anifrolumab (ANF, 2.5 µg/mL), then intracellular staining of IL-1 was performed. Representative contour plots and cumulative analysis of the percentage of IL-1β producing cells are shown, in unstimulated condition (**A**) or LPS-stimulated cells (**B**). Data are from 5 donors. Bars represent means and SD. *P < 0.05, ***P < 0.001, by 1way ANOVA with Tukey’s multiple comparisons test

Even in the absence of LPS stimulation, monocytes cultured with lipid-replete serum produced substantial amounts of IL-1β, whereas both IFN exposure and lipid deprivation strongly reduced IL-1β production. Notably, treatment with either a geranylgeranyl transferase inhibitor (GGTi) or the anti-IFNAR antibody anifrolumab (ANF) partially restored IL-1β production under LPDS conditions (Fig. 5A), indicating that lipid restriction suppresses IL-1β production in a prenylation- and IFN-dependent manner. In LPS-stimulated monocytes, both IFN exposure and LPDS significantly reduced IL-1β levels compared to NS; however, in this setting, the suppression was not reversed by GGTi or ANF (Fig. 5B). Interestingly, TNF and IL-6 production were differentially modulated by IFN. In monocytes not exposed to LPS, IFN significantly increased TNF and IL-6 production, whereas lipid deprivation did not reproduce this effect (Suppl. Figure 6–7).

Taken together, these findings indicate that lipid restriction specifically suppresses IL-1β production in monocytes through a prenylation-dependent amplification of IFN signaling. Several mechanisms may explain how IFN signaling limits IL-1β induction. Type I IFNs are known to suppress mitochondrial oxidative metabolism, thereby reducing mitochondrial ROS (mtROS), a potent activator of the inflammasome [8, 41]. We therefore investigated whether lipid restriction affects mitochondrial function.

**Fig. 6:**
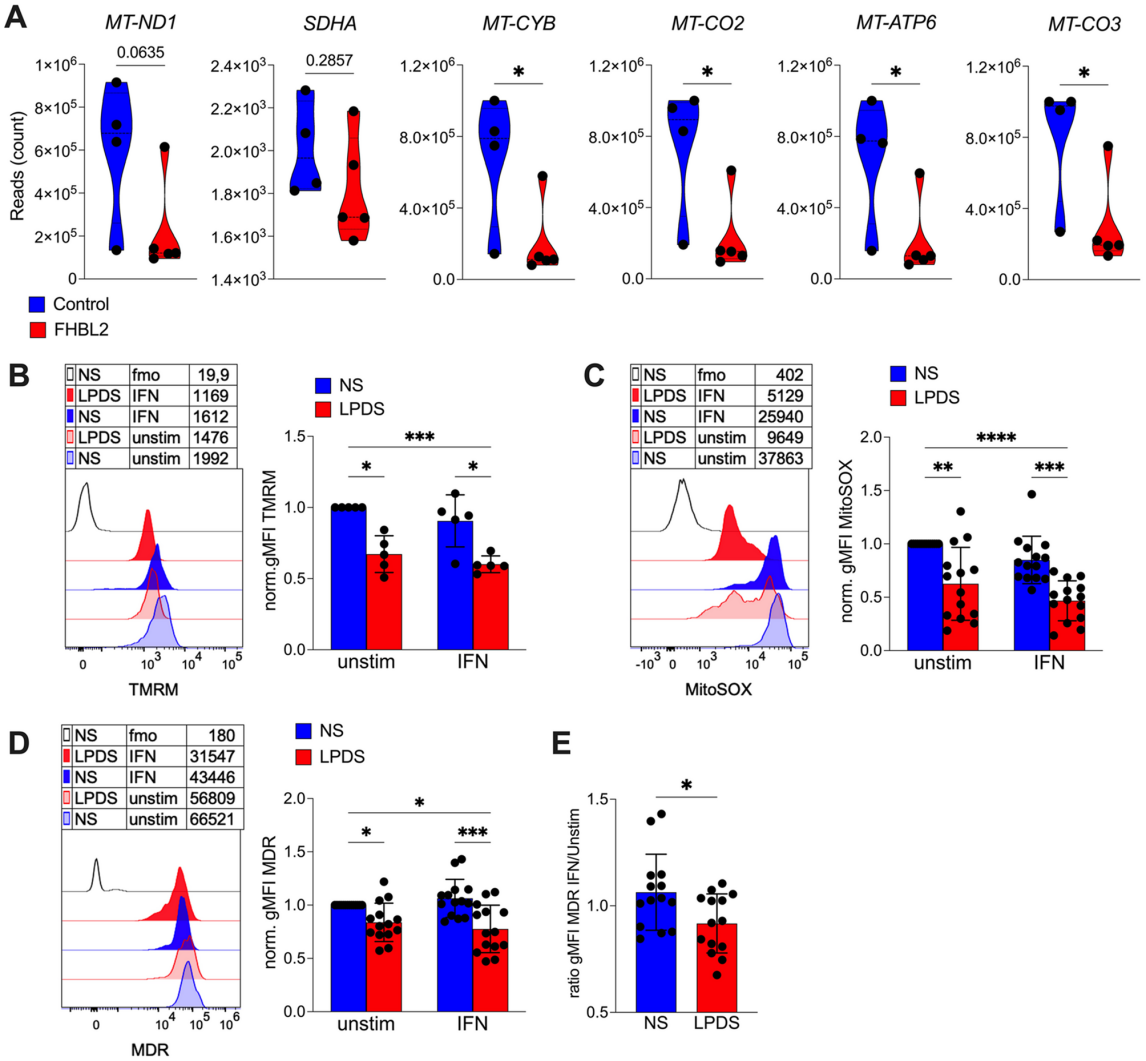
Reduced mitochondrial metabolism in lipid deprived serum in vitro and ex vivo in PBMCs of FHBL2. **A** Relative abundance of genes encoding for the mitochondrial ETC in the gene expression analysis of PBMCs from FHBL2 and control subjects. *P < 0.05 by Mann-Whitney test. **B-E** Monocytes from healthy controls were cultured 18 h with NS or LPDS, alone or plus IFN (40.000 IU/ml). Histogram overlays of gMFI, and the cumulative analysis of normalized gMFI (towards the NS/unstim sample), are shown, for TMRM (**B**), MitoSOX (**C**) and Mitotracker Deep Red (MDR, **D**). Data are from 5 donors (**B**), or 14 donors from 4 independent experiments (**C**-**D**). *P < 0.05, **P < 0.01, ***P < 0.001, ****P < 0.0001 by 2way ANOVA with Tukey’s multiple comparisons test. (**E**) Ratio of the MDR gMFI in IFN versus unstimulated cells. *P < 0.05 by Wilcoxon test

RNA-seq analysis of PBMCs from FHBL2 subjects revealed that nearly all genes encoding subunits of the electron transport chain (ETC) complexes were downregulated (with the exception of *MT-ND1* and *SDHA*) compared with controls (Fig. 6A). Consistently, lipid-deprived monocytes displayed a significant reduction in mitochondrial membrane potential, as measured by tetramethylrhodamine methyl ester (TMRM) staining (Fig. 6B), decreased mtROS levels as detected by MitoSOX (Fig. 6C), and reduced mass of active mitochondria as measured by MitoTracker Deep Red (MDR) (Fig. 6D). Notably, IFN further suppressed mitochondrial activity, and this inhibitory effect was significantly stronger in LPDS-treated cells compared to NS-treated cells (Fig. 6E), suggesting a synergistic interaction between lipid deprivation and IFN signaling in constraining mitochondrial function. Thus, lipid restriction reduces mtROS production, removing a key trigger for inflammasome activation.

## Discussion

Here, by investigating the immunometabolic changes in FHBL2, we uncovered a novel circuit linking the hypolipidemia typical of this condition to the suppression of pro-atherogenic inflammation. This circuit involves the monocyte-intrinsic induction of isoprenoid synthesis, amplification of the type I IFN response, and suppression of mitochondrial metabolism, ultimately resulting in inflammasome inhibition.

A spontaneous IFN signature emerged in PBMCs from FHBL2 subjects. This finding mirrors the observation of a suppressed IFN signature in monocytes from FH patients, which was restored by lipid-lowering therapy [17]. An IFN signature is associated with several autoimmune diseases, such as lupus [2]. Moreover, chronic IFN signaling is associated with impaired control of viral infections and cancer due to refractoriness to acute IFN stimulation [42]. To date, no evidence has suggested an increased risk of autoimmune disease, chronic viral infections, or impaired vaccine responses in FHBL2 individuals. However, FHBL2 is an ultra-rare condition, and current clinical data are limited to a small number of individuals [28]. Therefore, the absence of such reports should be interpreted with caution and does not exclude the possibility that these associations may emerge in larger cohorts. Nevertheless, we propose that the IFN signature represents a protective response in FHBL2 individuals against pro-atherogenic inflammasome activation, potentially contributing to their reduced cardiovascular risk [19].

Monocytes from FHBL2 subjects exhibited a significantly lower content of lipid droplets, as assessed by Bodipy labeling. The intracellular lipid content strongly and positively correlated with plasma concentrations of triglycerides, total cholesterol, LDL, and HDL. This result indicates that intracellular lipid storage closely reflects the availability of extracellular plasma lipoproteins. This is not an exclusive feature of monocytes, as we observed a similar correlation for intracellular lipid content in CD4 T cells from FHBL2 individuals [21]. However, monocytes display the highest Bodipy staining among total PBMCs (not shown), suggesting that they are particularly sensitive to variations in the plasma lipid profile.

In FHBL2 individuals, we also found that NK cell–related signatures were underrepresented in PBMCs. We were not able to determine whether NK cells were reduced in number, impaired in function, or redistributed from the blood to tissues. While it is well established that lipid overload impairs NK cell function [43], the effects of hypolipidemia on this immune population have not been investigated and warrant future study.

In vitro, we found that exogenous IFN increased CD38 expression and decreased CD14 expression on monocytes. These data are consistent with previous evidence showing that IFN enhances CD38 expression in various cell types [44, 45] and suppresses CD14 expression via the ISG Ly6E [46]. Conversely, monocytes from FHBL2 subjects, which display a spontaneous IFN signature, exhibited a CD38^low^ CD14^high^ phenotype. The discrepancy between in vivo and in vitro data may be explained by the fact that chronic and acute IFN exposure can have opposite effects on immune cell phenotype and function. However, due to the fragility of monocytes in culture, we could not directly test whether chronic, low-dose IFN exposure induces a CD38^low^ CD14^high^ phenotype in vitro. Other explanations for this discrepancy are also possible, as the CD38^low^ CD14^high^ phenotype may not be directly linked to IFN signaling but instead to other signals and metabolites. Indeed, several metabolites are differentially represented in plasma of FHBL2 subjects compared with controls [25]. Notably, CD38 ligation has been shown to induce the secretion of cytokines, including IL-1β, in human monocytes [47], suggesting that its modulation may directly contribute to the regulation of inflammatory responses. Regarding CD14, its internalization into endosomes has been shown to drive type I IFN production [48]. Therefore, it is plausible that the CD14^high^ phenotype in FHBL2 is associated with reduced receptor internalization, making it unlikely that enhanced IFN signaling in FHBL2 is driven by CD14 engagement.

In monocytes enriched from healthy donors and cultured in vitro under lipid restriction, we could reproduce not only the reduction in intracellular lipid content but also the enhanced sensitivity to IFN signaling. This result demonstrates a causal relationship between lipid starvation and IFN hypersensitivity in a monocyte-intrinsic manner. However, it should be noted that our in vitro model may not fully recapitulate all the features of circulating monocytes in FHBL2 subjects, who are chronically exposed not only to hypolipidemia but also to alterations in several other non-lipid metabolites that may influence their phenotype. For example, we previously demonstrated that FHBL2 subjects display elevated levels of the ketone body β-hydroxybutyrate [25], a metabolite with well-known anti-inflammatory properties mediated by its ability to suppress inflammasome activation [49]. Moreover, after a fat meal challenge, FHBL2 carriers displayed higher lactate, citrate, and acetate levels [25]. Notably, among the Free Fatty Acid Receptors (FFAR1–4), the gene encoding FFAR2 (GPR43), which specifically recognizes short-chain fatty acids, was significantly upregulated in PBMCs from FHBL2 subjects compared to controls (not shown). In human monocytes, FFAR2 engagement has been shown to inhibit multiple inflammatory cytokines, including IL-1β [50]. In mice, FFAR2 and FFAR3 protect against hypertension through immune-mediated mechanisms [51]. Overall, these findings suggest that, in vivo, a heightened response to these metabolites in FHBL2 subjects may contribute to redirecting monocyte functions toward anti-inflammatory and atheroprotective activity.

Our data parallel results obtained by others, showing that LDL overload specifically suppresses the IFN response in vitro in macrophages [17]. However, in our setting, complementation with exogenous LDL only partially rescued the control of the IFN response. This finding may suggest that different lipoproteins in the serum cooperate in this process through distinct activities. HDL is unlikely to be part of this mechanism. Others have shown that cholesterol removal by HDL can inhibit the IFN response induced by TLR4 triggering in human macrophages [52, 53]. However, we observed that both FHBL2 monocytes ex vivo and LPDS-exposed monocytes in vitro displayed a lower, not higher, intracellular lipid content, which is not compatible with reduced cholesterol efflux due to HDL depletion.

Under LPDS exposure, we observed a slight increase in ISG expression in monocytes, even in the absence of exogenous IFN. This suggests that lipid deprivation may not only enhance IFN responsiveness but also induce a spontaneous, though very low, IFN release by monocytes. In line with this possibility, we detected very low IFNα production in monocytes, which was slightly increased under LPDS conditions (not shown). However, monocytes are not professional IFNα producers, and it is therefore possible that, in vivo, other cells represent relevant sources of IFN.

In both regulatory T cells [21] and monocytes, we showed that lipid restriction activates the cell-intrinsic mevalonate pathway, which in turn boosts immune signaling through prenylation. The role of prenylation in enforcing Treg signals has also been demonstrated in other contexts [54]. Here, we identify prenylation as a metabolic amplifier of IFN responses. Our findings are consistent with previous studies by others [55], showing that statins attenuate IFN production and signaling in murine macrophages. Similar to our results, statins reduced STAT1 phosphorylation, an effect that was reversed by supplementation with geranylgeranyl pyrophosphate [55]. Isoprenoids are required for the prenylation of small GTP-binding proteins such as Rho and Rac GTPases. RhoA expression has been reported to correlate positively with the IFN signature in lupus patients and to directly promote STAT1 phosphorylation downstream of IFNAR [56].Whether RhoA prenylation and function are enhanced in FHBL2-derived monocytes and thereby contribute to the amplification of IFN signaling in these subjects remains to be determined.

In vitro, lipid starvation and IFN exposure were equally effective in inhibiting IL-1β production in monocytes. Notably, this effect was weaker when cells were stimulated with LPS, indicating that strong TLR4 engagement can counteract this regulatory circuit. It should also be noted that, under LPS stimulation in vitro, not only the effects of anifrolumab or GGTi but also the inhibitory activity of IFN or LPDS on IL-1β production were weaker than in unstimulated cells. Therefore, this pathway may be overall less relevant under conditions of strong TLR4 activation. Whether other key components of the inflammasome pathway are involved remains an open question.

Downstream of IFN signaling, several mechanisms can mediate IL-1β inhibition. Others have shown that the ISG CH25H generates a metabolite that directly suppresses inflammasome activation [9]. In our setting, however, we found that *CH25H* was not among the ISGs modulated in vivo in FHBL2 subjects and was not significantly induced by IFN in vitro (not shown). Instead, we observed that multiple mitochondrial features—including active mass, mtROS production, and membrane polarization—were all significantly reduced under lipid restriction. Mitochondrial respiration and mtROS release are repressed by IFN signals [41], yet both are key events in inflammasome activation [8, 57]. In our study, we did not demonstrate that restoring mitochondrial function in lipid-deprived monocytes would rescue IL-1β production. However, the link between mitochondrial oxidative metabolism and inflammasome activation is well established in the literature [8, 57, 58]. Based on our findings, we propose a model in which hypolipidemia causes intracellular cholesterol scarcity, thereby stimulating isoprenoid synthesis within immune cells. This leads to enhanced prenylation of signaling molecules and potentiation of type I IFN responses, which repress oxidative metabolism and ultimately inhibit IL-1β production. At the same time, lipid restriction reduces mitochondrial metabolism, further contributing to inflammasome repression. Overall, this immunometabolic circuit links the external lipid environment to innate immune signaling and reveals mechanisms that may underlie cardioprotective innate immune responses.

These findings may also have relevance beyond the context of rare genetic conditions, particularly in light of the emerging use of ANGPTL3 inhibitors and other lipid-lowering therapies in the general population. Indeed, our results raise new hypotheses regarding the impact of lipid-lowering therapies on innate immunity. Therapies such as PCSK9 inhibitors have been shown to increase LDLR expression in macrophages and amplify IFN responses [59], and ANGPTL3 inhibitors [20] may exert similar effects. It remains to be determined whether these pharmacologic strategies modulate the IFN signature in vivo and whether this contributes to their cardioprotective effects.

This study has some limitations that should be acknowledged. First, FHBL2 is an ultra-rare condition, and the number of individuals available for ex vivo analyses was necessarily limited. Nonetheless, our findings were consistent across independent samples and were further supported by in vitro models that reproduced key phenotypes—namely, reduced intracellular lipid content and enhanced IFN sensitivity—measured with multiple approaches and experimental readouts at both gene and protein levels. Second, the mechanistic insights presented here were primarily obtained from monocytes cultured under lipid-deprived conditions. While these models provide strong evidence of causality, future studies will be required to determine whether similar mechanisms operate in vivo during therapeutic ANGPTL3 inhibition. Even within the in vitro setting, some mechanistic links remain to be clarified. We did not demonstrate that RhoA is the key prenylation target responsible for IFN signal amplification in our model. Furthermore, we could not determine whether cholesterol content and intracellular distribution were indeed remodeled, nor whether lipoproteins other than LDL could efficiently compensate for lipid deprivation.

## Supplementary Information

Below is the link to the electronic supplementary material.

Supplementary Material 1

## Data Availability

All data generated or analyzed during this study are included in this published article. The RNA-Seq accompanying this paper are available through NCBI’s Gene Expression Omnibus (GEO) repository, under accession number GSE307462.
