# Supplementary material for: Lipid deprivation amplifies type I IFN responses in monocytes through prenylation: insights from familial combined hypolipidemia type 2
Source: J Transl Med. 2025 Nov 25;24:3. doi: 10.1186/s12967-025-07448-5 (PMC12764162; doi:10.1186/s12967-025-07448-5)
Supplement: Supplementary file 1 — Supplementary Material 1 [file 12967_2025_7448_MOESM1_ESM.docx]

***Supplementary material***


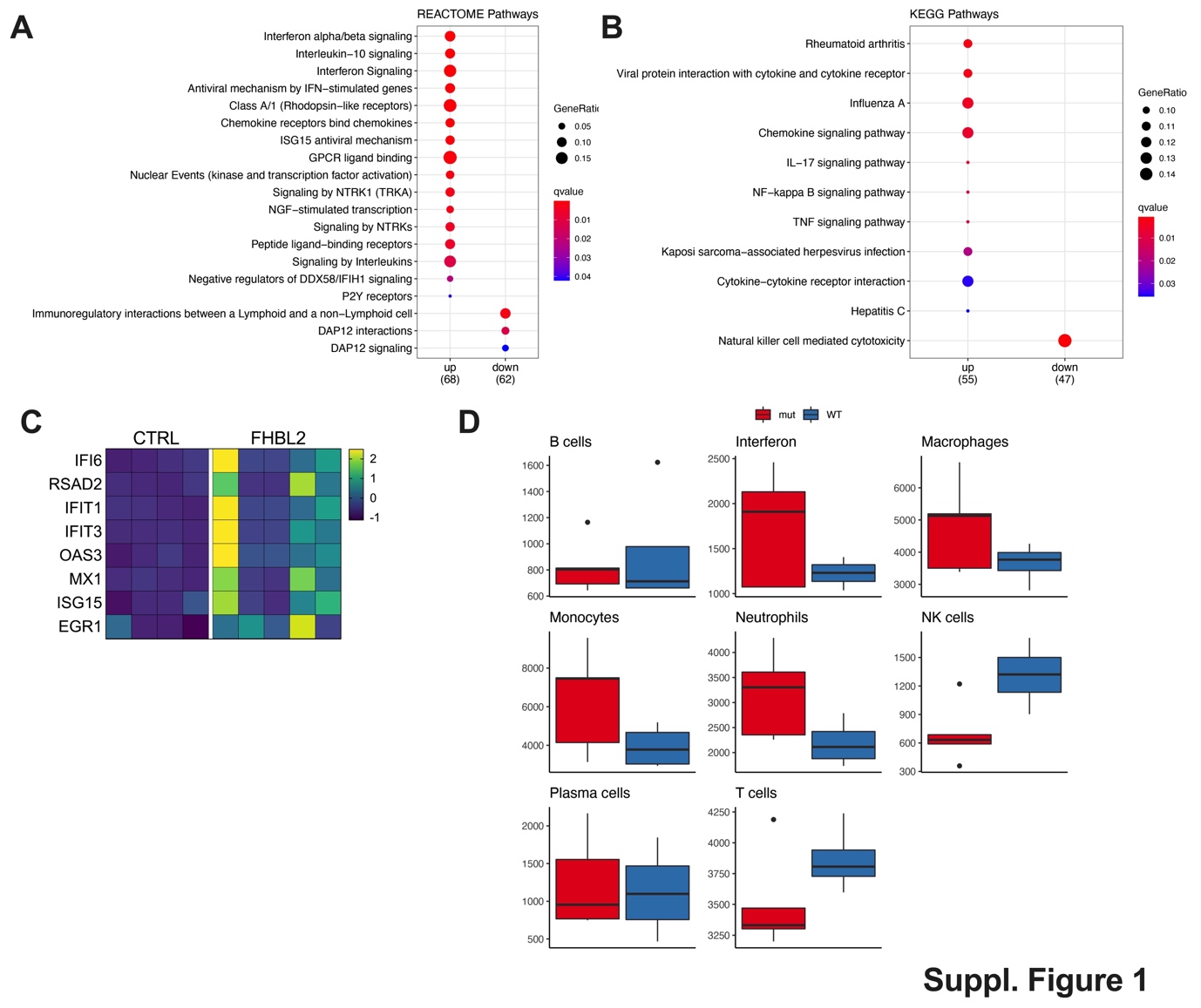


***Supplementary Figure 1. Immune signatures in PBMCs of FHBL2 homozygous subjects***

PMBCs from FHBL2 homozygous subjects (n=5) and age- and sex-matched non mutated controls (n=4) were analyzed by bulk RNAseq analysis.

**A-B** REACTOME (A) and KEGG (B) pathways analysis of the DEGs in PBMCs of FHBL2 and controls.

**C** Heatmap of the relative abundance (as the z-score) of selected ISG transcripts.

**D** Enrichment of selected signatures of immune cells in FHBL2 (mut) and controls (wt).

***
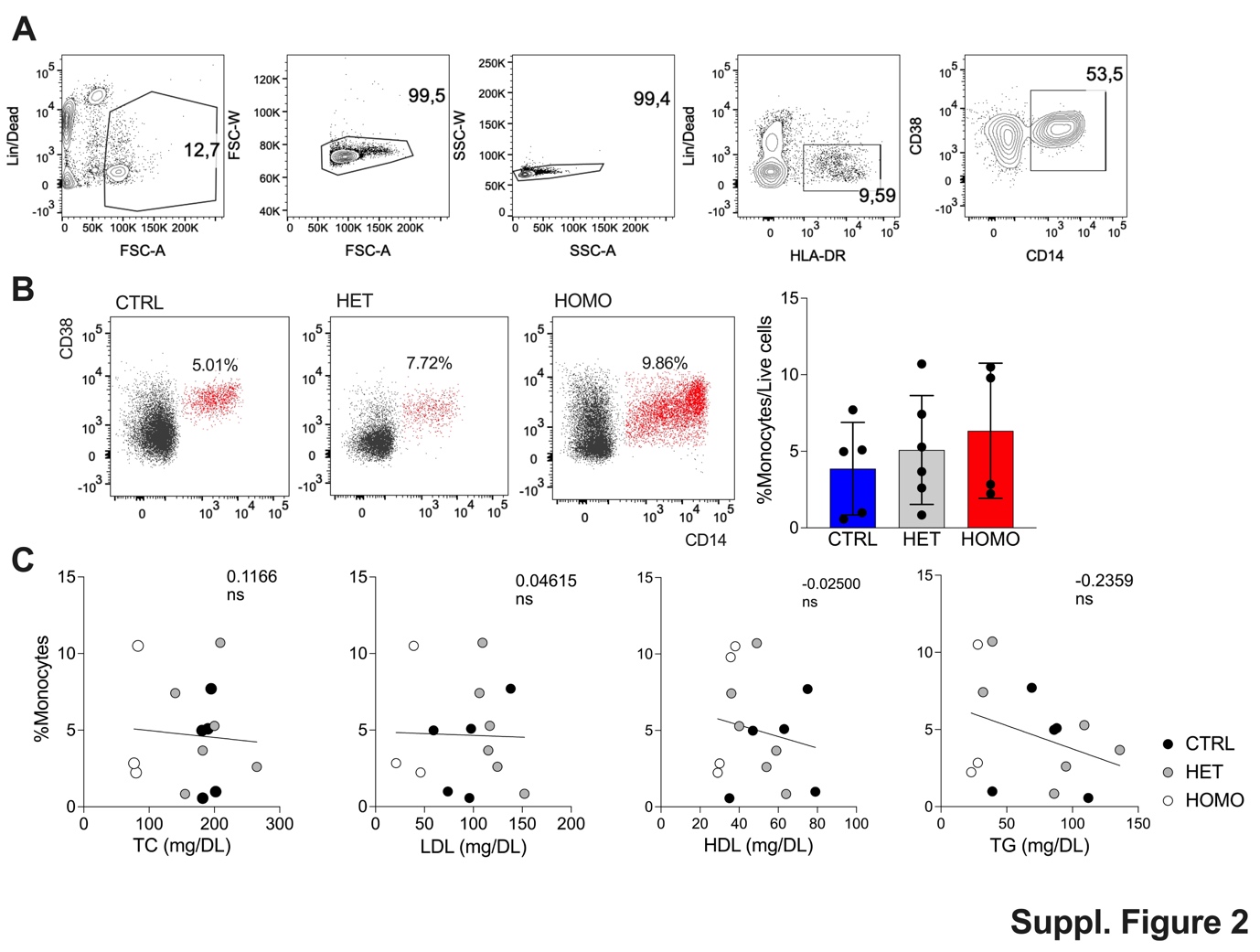
Supplementary Figure 2. Trend for higher monocyte percentage in homozygous FHBL2 subjects***

**A** Gating strategy to identify monocytes. Briefly, among live single cells, HLA-DR+ Lineage (CD4/8/19/56)- were selected, and within this subset the monocytes were gated as CD14+ CD38+ cells.

**B** Representative dot plots of the percentage of CD38+ CD14+ monocytes (in red) among live single cells (grey) in controls, heterozygous and homozygous FHBL2. The numbers in each plot represent monocyte percentage. Data are from 4 homozygous, 6 heterozygous and 5 controls. Each dot represents a single subject. Bars represent means and SD.

**C** Correlation between monocyte percentages and plasma concentrations of total cholesterol (TC), low-density lipoprotein (LDL), high-density lipoprotein (HDL), and triglycerides (TG) in each subject. Numbers indicate the nonparametric Spearman r coefficient and the p value. The line in each plot represents the simple linear regression. ns, not significant.

***
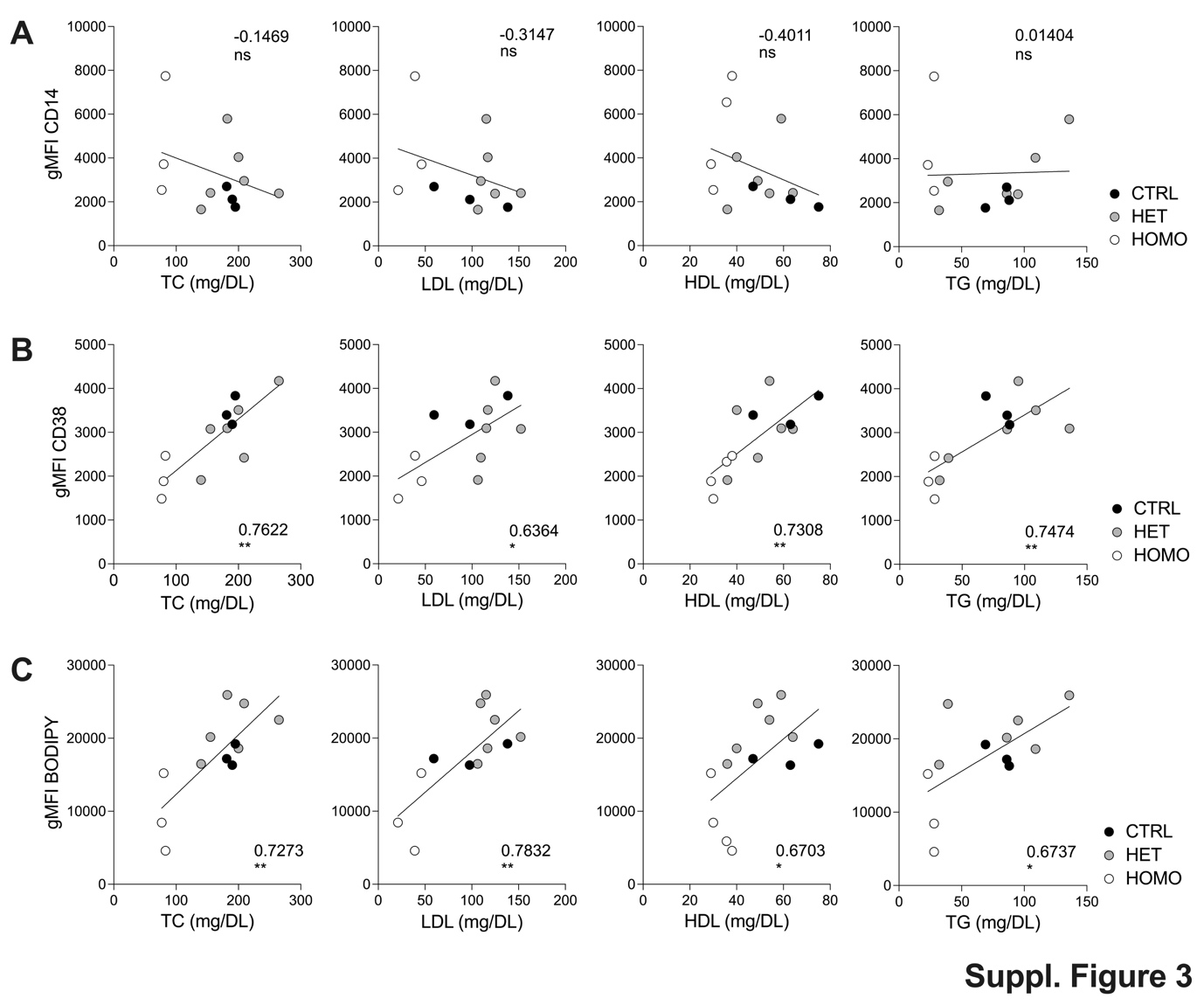
 Supplementary Figure 3.*** ***Positive correlation between plasma lipids and monocyte profile***

Correlation between the extent (measured as the gMFI) of CD14 expression (**A**), CD38 expression (**B**) and Bodipy level (**C**), versus the plasma concentrations of total cholesterol (TC), low-density lipoprotein (LDL), high-density lipoprotein (HDL), and triglycerides (TG) in each subject. Data are from 3-4 homozygous, 6 heterozygous and 3 controls. Numbers indicate the nonparametric Spearman r coefficient and the p value. The line in each plot represents the simple linear regression. *P<0.05, **P<0.01, by Spearman correlation. ns, not significant.

***
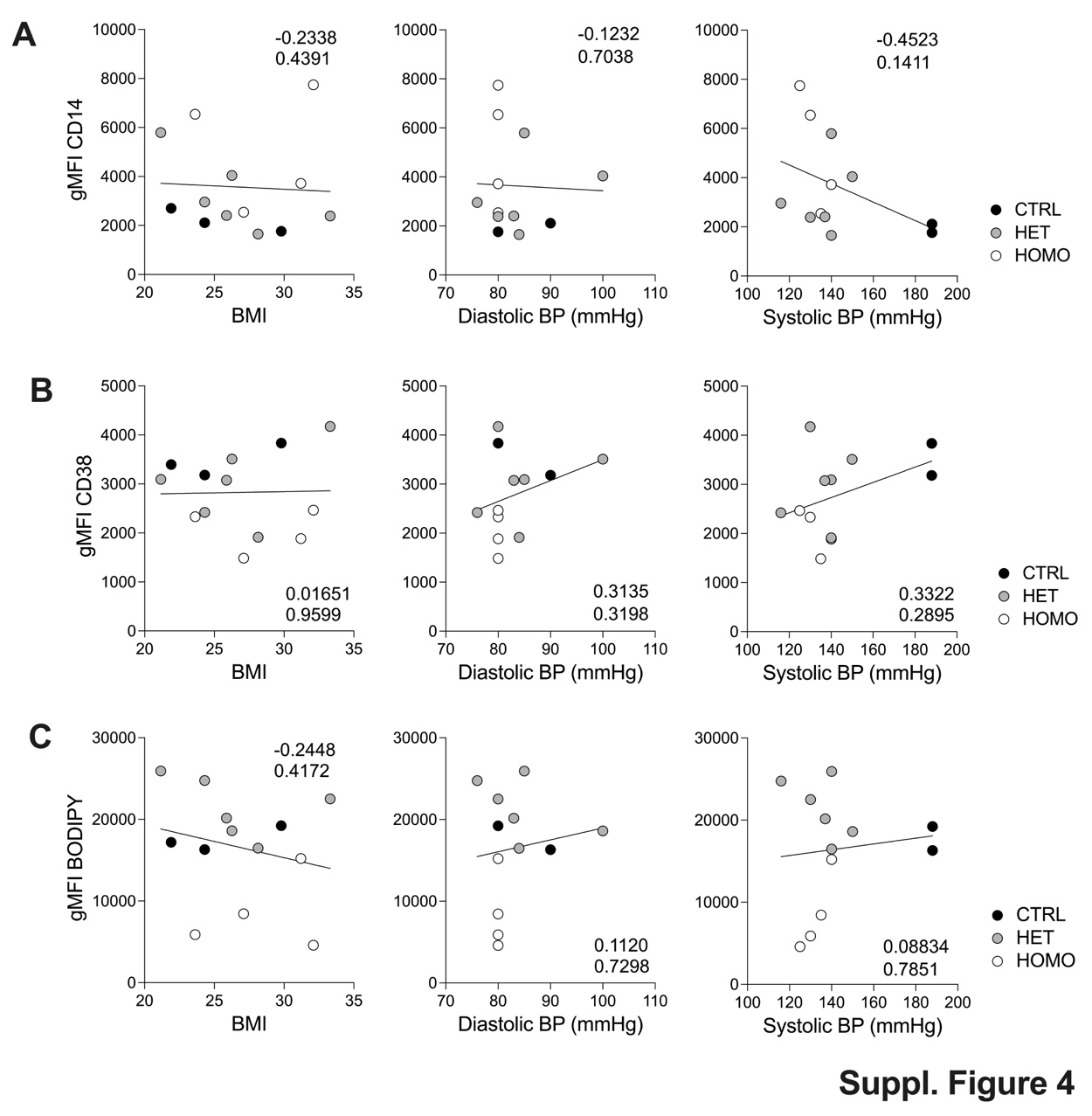
***

***Supplementary Figure 4. Correlations between monocyte profile and cardiometabolic parameters***

Correlation between the extent (measured as the gMFI) of CD14 expression (A), CD38 expression (B) and Bodipy level (C), versus the body-mass index (BMI), the diastolic and the systolic blood pressure (BP) in each subject. Data are from 4 homozygous, 6 heterozygous and 2-3 controls. Numbers indicate the nonparametric Spearman r coefficient and the p value. The line in each plot represents the simple linear regression.

***
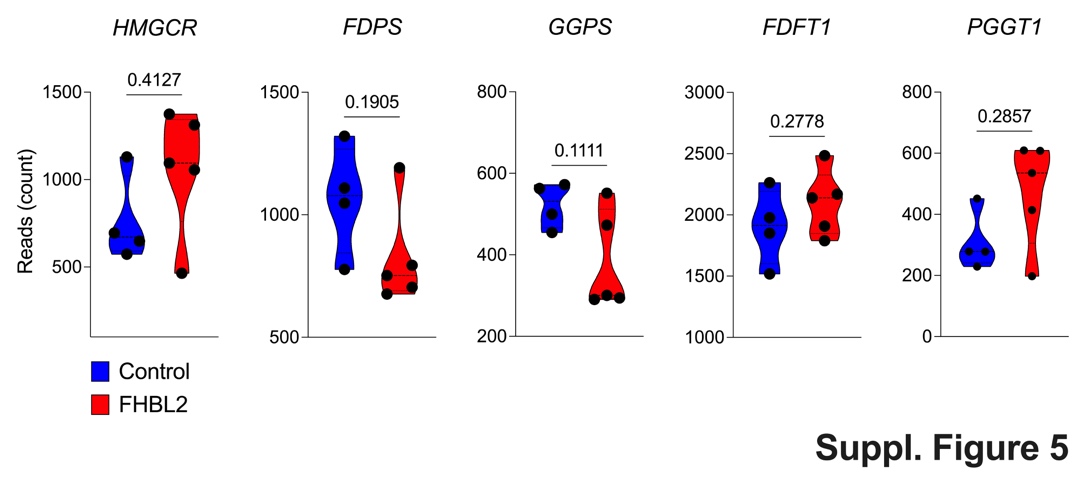
***

***Supplementary Figure 5. Expression of genes of the mevalonate pathway ex vivo in FHBL2***

Relative abundance of genes encoding for mevalonate pathway in the gene expression analysis of PBMCs from FHBL2 and control subjects. Numbers indicate the P values, by Mann-Whitney test.

***
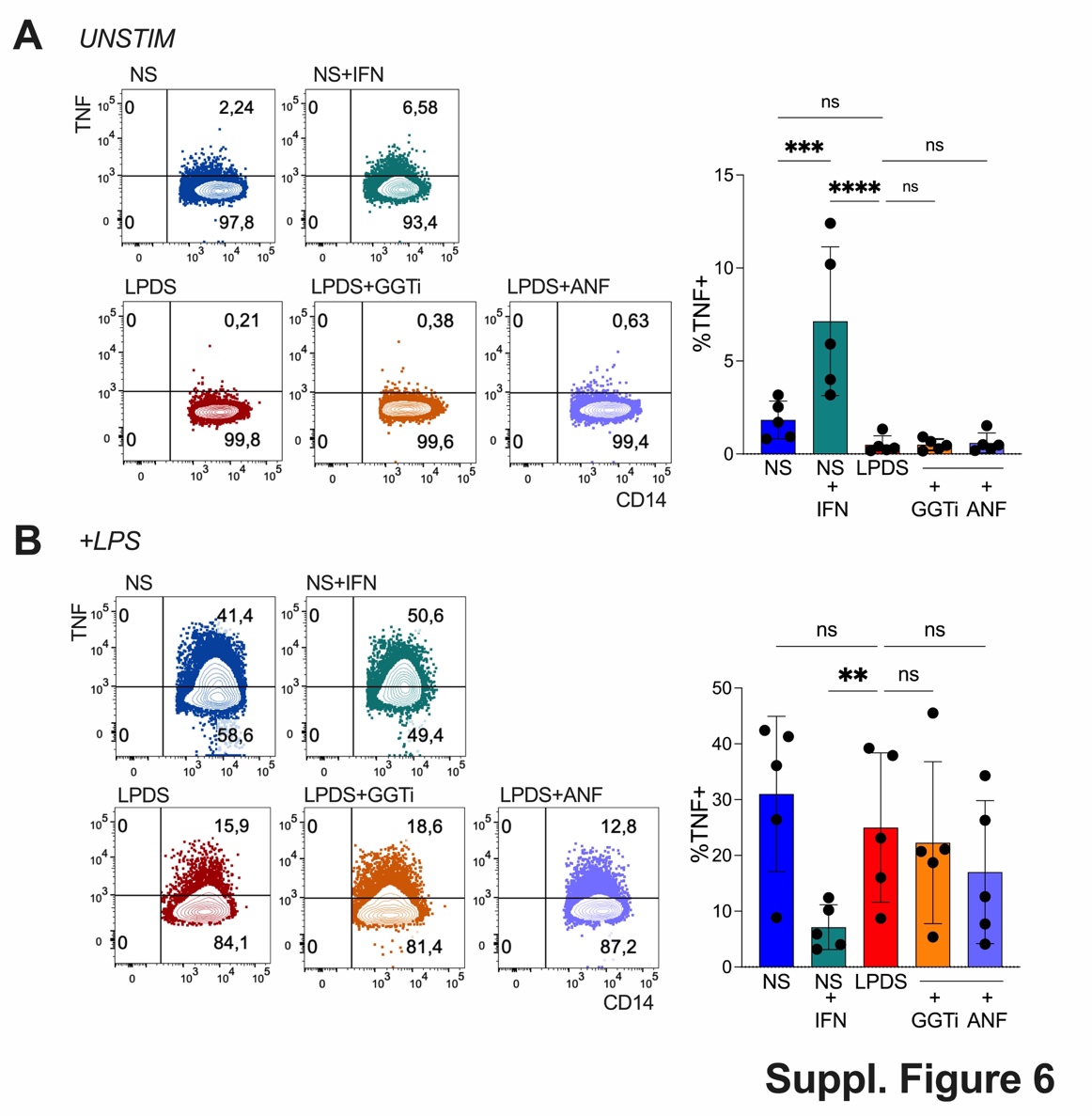
***

***Supplementary Figure 6.*** ***TNF production is not affected by LPDS***

Monocytes, cultured 18 hrs with NS or LPDS, were left unstimulated or stimulated with LPS (100 ng/mL), IFN (40.000 IU/mL), GGTi (30 μM) or anifrolumab (ANF, 2.5 μg/mL), then intracellular staining of TNF was performed.

Representative contour plots and cumulative analysis of the percentage of TNF producing cells are shown, in unstimulated condition (A) or LPS-stimulated cells (B).

Data are from 5 donors. Bars represent means and SD. **P<0.01, ***P<0.001, ****P<0.0001, by 1way ANOVA with Tukey’s multiple comparisons test.

***
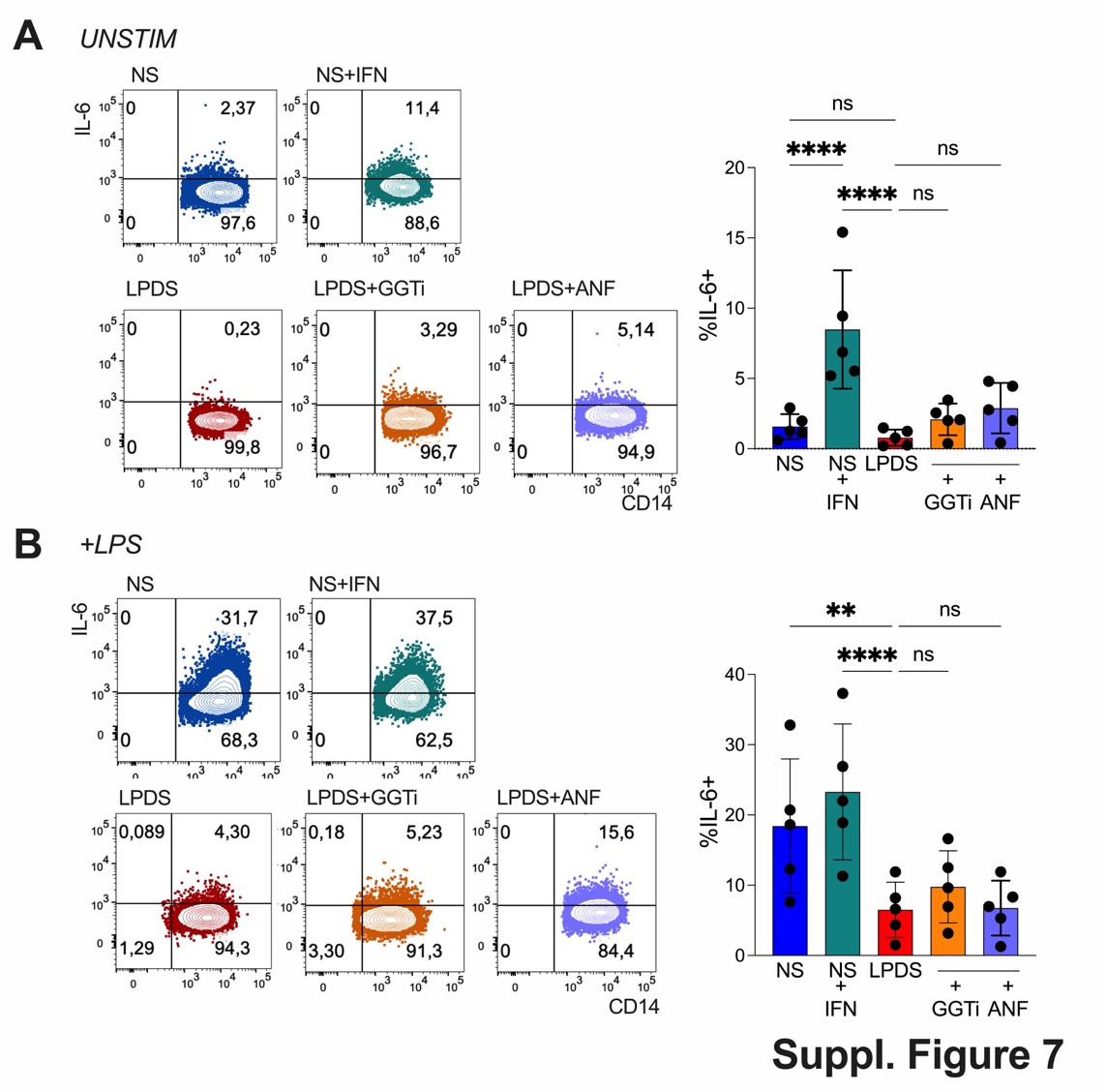
***

***Supplementary Figure 7.*** ***IL-6 production is not affected by LPDS***

Monocytes, cultured 18 hrs with NS or LPDS, were left unstimulated or stimulated with LPS (100 ng/mL), IFN (40.000 IU/mL), GGTi (30 μM) or anifrolumab (ANF, 2.5 μg/mL), then intracellular staining of IL-6 was performed.

Representative contour plots and cumulative analysis of the percentage of IL-6 producing cells are shown, in unstimulated condition (A) or LPS-stimulated cells (B).

Data are from 5 donors. Bars represent means and SD. **P<0.01, ****P<0.0001, by 1way ANOVA with Tukey’s multiple comparisons test.
